# Meta-Analysis of the Expansion in the Field of Structural Biology of ABC Transporters

**DOI:** 10.34133/2022/9806979

**Published:** 2022-09-08

**Authors:** Soomi Kim, Teena Bajaj, Cole Chabon, Eric Tablante, Tatyana Kulchinskaya, Tae Seok Moon, Ruchika Bajaj

**Affiliations:** ^1^Stem Cell Technology Certificate Program, City College of San Francisco, USA; ^2^Biotechnology Certificate Program, City College of San Francisco, USA; ^3^Comparative Biochemistry Program, University of California Berkeley, USA; ^4^Department of Energy, Environmental and Chemical Engineering, Washington University in St. Louis, USA; ^5^Department of Bioengineering and Therapeutics Sciences, University of California San Francisco, USA

## Abstract

ABC transporters are molecular machines which power the solute transport using ATP hydrolysis. The structural biology of ABC transporters has been exploding for the last few years, and this study explores timelines and trends for various attributes such as structural tools, resolution, fold, sources, and group leaders. This study also evidences the significance of mammalian expression systems, advancements in structural biology tools, and the developing interest of group leaders across the world in the remarkably progressing field. The field started in 2002 and bloomed in 2016, and COVID years were really productive to the field. Specifically, the study explores 337 structures of 58 unique ABC transporters deposited in the PDB database from which P-glycoprotein has the largest number of structures. Approximately, 62% of total structures are determined at the resolution of 3-4 Å and 53% of structures belong to fold IV type. With progressive advancements in the field, the field is shifting from prokaryotic to eukaryotic sources and X-ray crystallography to cryoelectron microscopy. In the nutshell, this study uniquely provides the detailed snapshot of the field of structural biology of ABC transporters with real-time data.

## 1. Introduction

ATP-binding cassette (ABC) transporters are members of a large superfamily of transporters which bind and hydrolyze ATP to move a diverse library of substrates across the membrane [1–3]. ABC transporters function as importers, exporters, or flippases (flip lipids from one leaflet of membrane to the other) or extractors (extract out the lipid from the membrane) [4]. These transporters, playing roles in key physiological processes, are ubiquitously present in all organisms and represent a significant class of molecules to study with respect to structure and function [5]. Consequently, these are associated with a multitude of human diseases and clinical complications including but not limited to cystic fibrosis, macular degeneration, retinitis pigmentosa, and multidrug resistance in cancer [6, 7]. ABC transporters mainly consist of two transmembrane domains (TMDs) forming the pathway for the substrate in the membrane and two nucleotide-binding domains (NBDs) containing conserved sequences to bind and hydrolyze ATP in order to power the solute transport across the membrane [2, 8]. Some ABC transporters also utilize accessory proteins like high-affinity solute-binding protein which sequester the solute using high-affinity binding site and transfer it to the transmembrane domains [1, 2].

**Figure 1:**
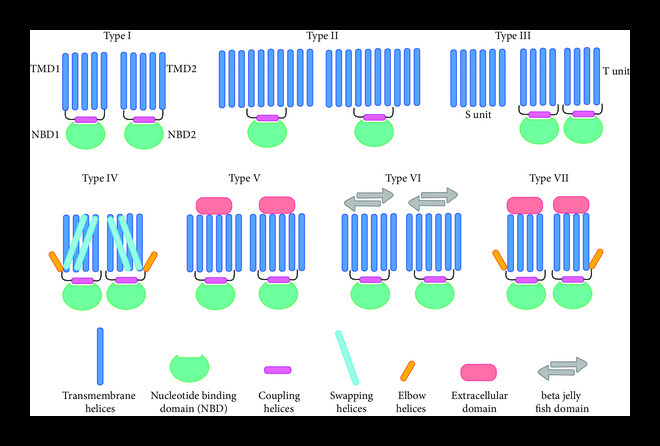
Seven structural folds in structurally studied ABC transporters.

Advancements in structural tools such as X-ray crystallography and cryoelectron microscopy (cryo-EM) in combination with improvements in mammalian expression systems have revolutionized the field of structural biology of ABC transporters and progressed our understanding towards their mechanism [9, 10]. They are viewed as using alternating access mechanism, i.e., undergoing transition in between inward-facing and outward-facing conformations during solute transport across the membrane [1, 2, 11]. Structural biology of ABC transporters certainly contributed towards the discovery and defining of different structural folds based on specific structural features such as number of transmembrane helices, swapping of helices across TMDs, presence of elbow helices, or presence of large extracellular domains or beta-jellyroll-like domains, translocation pathway, and interaction of coupling helices from TMDs with NBDs (Figure 1) [4, 12]. Different folds, represented differently in prokaryotes and eukaryotes, also correlate with their function as an importer, exporter, flippase, or extractor [4].

Seven categories of structural folds have been defined in the structural biology of ABC transporters [4, 12] (Figure 1). Fold I or type I importer is the simplest category of folds in ABC transporters, which contain at least 5 transmembrane helices in each of their TMDs [13]. These types of transporters are mostly present in prokaryotes and transport different types of sugars, amino acids, and peptides. One of the most studied type I importer is *Escherichia coli* maltose transporter, which transports maltose or maltodextrins from periplasmic space to the cytoplasmic side [14]. Like type I importer, type II importers also transport various substrates including vitamin B_12_ (cobalamin), heme, molybdate, and metal chelates, from outside to inside of bacterial cells. However, TMDs in fold II transporters consists of 10 transmembrane helices [15]. Similar to type I importers, type II importers also use substrate-binding protein (SBP) for solute transport and coupling helices for TMD-NBD interdomain communication [13], albeit with the higher affinity for solutes than type I transporters. Type III importers, also known as ECF (energy coupling factor) transporters are predominantly found in prokaryotes and transport micronutrients, Ni^2+^, Co^2+^ and tryptophan across the cell membrane [13]. Unlike conventional ABC transporters, ECF transporters, such as LbECF and CbiMNQO, consists of two structurally and functionally unrelated TMDs, S and T, which contain 6 and 4-8 transmembrane helices, respectively. The S component binds substrate in periplasm and transport it to the T component which extends into the cytoplasm via coupling helices and communicates with NBDs [16]. Next, type IV transporters, represented both in prokaryotes as well as in eukaryotes, are defined by two TMDs containing 6 transmembrane helices, swapped helices, and elbow helices to interact with the membrane bilayer [17]. Some of the examples for type IV fold include Sav1866, MsbA, and TmrAB in prokaryotes and P-glycoprotein, MRP1, and TAP1/TAP2 in eukaryotes. Although type V and VI are also defined by two TMDs, each consisting of 6 transmembrane helices, no swapping of helices exists among TMDs. Nevertheless, both type IV and V contain elbow helices. Additionally, type V and VI contain large extracellular domains and beta-jellyroll domains, respectively. On the other hand, the type VII fold are defined by 4 transmembrane helices in each TMD, elbow helices, and large periplasmic domains. While type V fold is represented by WzmWzt in prokaryotes and ABCA1 and ABCG5/8 in eukaryotes, type VI and VII are represented only in prokaryotes by LptB_2_FG and MacB, respectively.

The explosion of structures of ABC transporters in the Protein Data Bank (PDB) database in the last decade intrigued us to comprehend the field of structural biology of ABC transporters. The course of discovery of these structures is studied during time period spanning last 20 years, i.e., from the beginning (2002) of determination of the first structure of an ABC transporter to now (May 2022) [12, 18]. This study collectively provides a subset of PDB database specifically focused on ABC transporters with a list of structurally studied ABC transporters and the related information. The purpose of this article is to gain a better understanding on the continuously evolving and growing nature of the field of structural biology of ABC transporters.

## 2. Data Mining and Analysis

**Figure 2:**
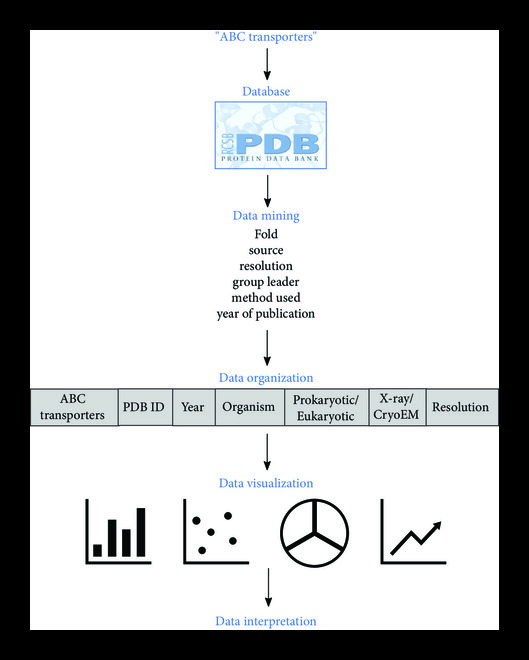
Workflow to study the expansion of structural biology of ABC transporters.

The workflow of the study is depicted in Figure 2. The PDB database (https://rcsb.org), which enables open access to the accumulating knowledge of 3D structure, function, and evolution of biological macromolecules, was used to gather information regarding structures of ABC transporters [19, 20]. The PDB database was searched for structures of ABC transporters and a list of unique ABC transporters with deposited structures was prepared. For each unique ABC transporter, associated structures and the data regarding their relevant information (year of release or publication of the structure, method to determine the structure, fold of the structure, source, function, resolution, and group leader whose lab published the structure) was mined in the form of a spreadsheet. Only published structures comprised of both TMDs and NBDs, as a part of our criteria, were included in the list. The list of unique ABC transporters as well as the associated data was updated as novel structures of ABC transporters were determined over time. The mined data was plotted on graphs in order to understand various trends or course of events in the rapidly moving field of structural biology of ABC transporters in the last two decades.

## 3. Unique ABC Transporters in PDB Database

**Figure 3:**
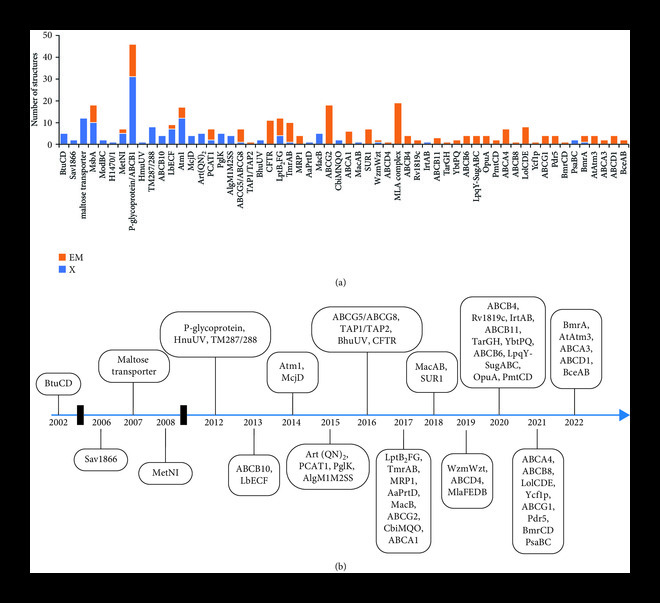
(a) Number of structures (y axis) for each unique ABC transporter (x axis). The height of bar indicates the number of structures determined for each unique ABC transporter. X and E are acronym for structural tools, X-ray crystallography and cryo-electron microscopy, respectively. Blue and orange color indicate the number of structures determined by X-ray crystallography and cryo-electron microscopy, respectively. (b) Timeline of unique ABC transporters structurally characterized for the first time.

The PDB database could retrieve 58 unique ABC transporters whose 337 structures have been determined either by X-ray crystallography or cryo-EM (Figure 3(a) and Table 1). A number of unique ABC transporters are being studied structurally every year, and the list of these ABC transporters is growing exponentially in PDB database (Figure S1). Structures of at least 19 and 28 of these unique ABC transporters were determined solely by either X-ray crystallography or cryo-EM, respectively (Figure 3(a)). However, structures for other eleven unique ABC transporters (MsbA, MetNI, P-glycoprotein (P-gp), LbECF, Atm1, PCAT1, ABCG5/ABCG8, LptB_2_FG, TmrAB, WzmWzt, and BmrA) were determined using both structural tools (Figure 3(a)). P-glycoprotein, the first eukaryotic ABC transporter whose corrected structure was first determined in 2012 and has been still seeking attention from group leaders in structural biology like Kasper Locher, has the largest number of structures, i.e., 46, in the PDB database (Figure 3(a)). Other eight unique ABC transporters including maltose transporter, MsbA, Atm1, CFTR, LptB_2_FG, TmrAB, ABCG2, and MlaFEDB have more than 10 structures in the PDB database (Figure 3(a)). Other ABC transporters mentioned in this study are a subject for further exploration via structural biology tools.

**Table 1:** Structurally studied unique ABC transporters and their function.

Year	ABC transporter	Function
2002	BtuCD	Translocate vitB12 from BtuF to cytosol
2006	Sav1866	Homolog of multidrug resistance transporter
2007	Maltose transporter	Imports maltose from periplasm to cytoplasm
2007	MsbA	Flip LPS-lipid molecule
2007	ModBC	Molybdate/tungstate transporter
2007	H1470/1	Molybdate/tungstate transporter
2008	MetNI	Transport methionine
2012	P-glycorprotein	Multidrug efflux pump
2012	HmuUV	Heme transporter
2012	TM287/288	Exporter for drugs like daunomycin
2013	ABCB10	Play a role in erythropoiesis and protection of mitochondria against oxidative stress
2013	LbECF	Transport vitB12 and folic acid
2014	Atm1	Heavy metal exporter
2014	McjD	Peptide tranporter
2015	Art(QN)_2_	Amino acid transporter
2015	PCAT1	Maturation protease and exporter to secrete antimicrobial polypeptides
2015	PglK	Flips lipid linked oligosaccharide
2015	AlgM1M2SS	Import alginate
2016	ABCG5/ABCG8	Excretes sterols
2016	TAP1/TAP2	Export out peptides
2016	BhuUV	Imports heme
2016	CFTR	Chloride channel
2017	LptB_2_FG	Exports liposaccharide from external leaflet and propels it along the filament
2017	TmrAB	Homolog of TAP1/TAP2, peptide exporter
2017	MRP1	Drug resistance pump
2017	AaPrtD	Polypeptide transporter for proteases - PrtA, B, C & D
2017	MacB	Extrudes a lot of peptides
2017	ABCG2	Exporter pump
2017	CbiMNQO	Cobalt transporter
2017	ABCA1	Effluxes phospholipids and cholesterol
2018	MacAB	Resistance to antibiotics
2018	SUR 1	Regulate insulin secretion in pancreatic beta cells
2019	WzmWzt	Translocate lipid-linked O-antigen
2019	ABCD4	Lysosomal cobalamin exporter
2019	MLA complex	Translocation of phospholipids
2020	ABCB4	Extrudes phosphatidylcholine into the bile canaliculi of the liver
2020	Rv1819c	Cobalamin transporter
2020	IrtAB	Siderophore-import machinery
2020	ABCB11	Bile salt exporter
2020	TarGH	Teichoic acid transporter
2020	YbtPQ	Siderophore uptake transporter
2020	ABCB6	Translocate toxic metals and anticancer drugs
2020	LpqY-SugABC	Uptake of nutrients like trehalose
2020	OpuA	Restores the cell volume by accumulating large amounts of compatible solute, gated by ionic strength
2020	PmtCD	Exporter of virulent peptide toxin
2021	ABCA4	Critical for photoreceptor function - rods and cones, transports all trans retinal, PE-retinal
2021	ABCB8	Involved in maturation of Fe-S and protects the heart from oxidative stress
2021	LolCDE	Tranport lipoprotein from gram negative bacteria
2021	Ycf1p	Yeast multidrug resistance protein, localizes to vacuolar membrane, functions as a glutathione conjugate transporter
2021	ABCG1	Excrete cholesterol in macrophages
2021	Pdr5	Confer resistance to anti-fungal compounds
2021	BmrCD	Multidrug efflux pump from bacillus
2021	PsaBC	Manganese importer
2022	BmrA	Conferring resistance to cervimycinC
2022	AtAtm3	Role in maturation of iron sulfur proteins, heavy metal detoxification, export glutathione derivatives
2022	ABCA3	Role in pulmonary surfactant biogenesis
2022	ABCD1	Fatty acid transporter
2022	BceAB	Sense and resist antimicrobial peptides

## 4. Timeline and Number of Structures Determined for Unique ABC Transporters

The timeline graph was delineated from the information regarding year in which the first structure of these unique ABC transporters was determined (Figure 3(b)). The first structure of an ABC transporter was determined in 2002 for a bacterial importer, BtuCD from *Escherichia coli* from the Rees group, which was followed by the structure of multidrug exporter Sav1866 in 2006 and has been followed with structures of many other ABC transporters until now in 2022 (Figure 3(b)). Although the field was in a lag phase until 2012, however, it continuously started exploring novel unique ABC transporters since then (Figure S1b). The year 2020, despite of being affected by a global pandemic COVID-19, has been one of the most productive years in terms of exploring 10 novel unique ABC transporters structurally mostly via using cryo-EM as a structural tool (Figure S1a). The year 2017, in which the second highest number, i.e., 8 unique ABC transporters were revealed structurally, acted as an explosion moment in the field of structural biology of ABC transporters (Figure S1a). Other prior years like 2015 and 2016 and the recent years 2021 and 2022 have also been prolific along the similar lines. The growing interest and productivity in the field among these years could be credited to advancements in expression systems for membrane proteins as well as advancement and applicability of cryo-EM to membrane proteins [21, 22].

## 5. Timeline Distribution with respect to Structural Tools, Source, Resolution, and Fold

**Figure 4:**
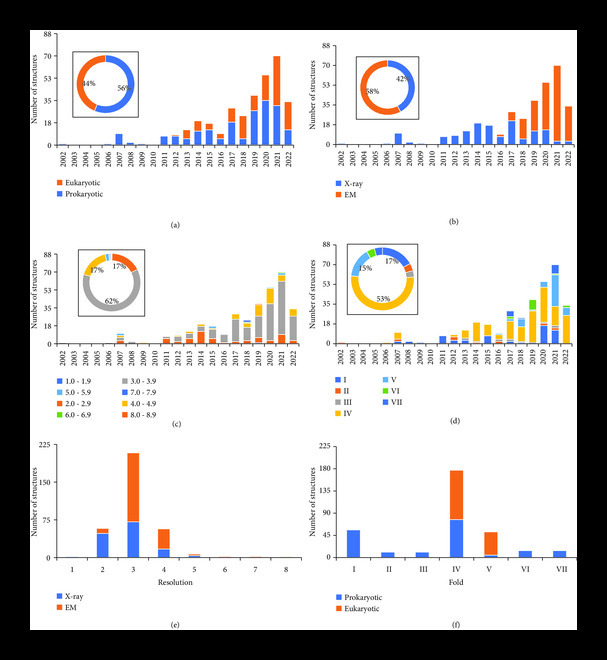
Timeline distribution of structures of ABC transporters (a) from prokaryotic and eukaryotic sources, (b) characterized by X-ray or EM, (c) determined at different resolutions (1-9 Å), (d) with different structural folds. Different categories are labeled and colored accordingly in the graph. (e) Distribution of structures of ABC transporters determined by X-ray and EM at different resolution. (f) Distribution of structures of ABC transporters from different sources with different structural folds. Different categories are labeled and colored accordingly in the graph. Insets are pie charts with percentages of these distributions. Percentages of minor distributions are not indicated.

Furthermore, the timeline distributions with respect to the information regarding sources, resolution, fold, and tools to determine structures were created to understand trends in the field and comprehend relationships among these aspects as well (Figure 4). The first structure of an ABC transporter was determined in 2002 from a prokaryotic source, i.e., *Escherichia coli* and the breakthrough in the field took advantage of a simple and low-cost recombinant bacterial expression system [23] (Figure 4(a)). Since then, only prokaryotic sources were used for determining structures in the field until 2012 when the first corrected structure of P-glycoprotein was determined from a eukaryotic source (PDB ID: 4F4C from *Caenorhabditis elegans*) (Figure 4(a)). After that, eukaryotic sources including *Homo sapiens* and *Mus musculus* have been very common in the field which can be credited to improvements in insect, yeast, and mammalian expression systems for membrane proteins [24–26]. Amazingly, mitochondrial ABC transporter, Atm1, has been determined from prokaryotic (*Novosphingobium aromaticivorans*) as well as eukaryotic (*Saccharomyces cerevisiae*) sources. As the usage of eukaryotic sources has been continuously increasing since 2012, the proportion of structures from prokaryotic sources (56%) is comparable to eukaryotic sources (44%) (Figure 4(a) inset). Figure 4(b) shows the timeline distribution for usage of structural tools (mainly X-ray crystallography and cryo-EM) to determine a number of structures in the field. It clearly shows that the art of X-ray crystallography was used at the initial stages of the field to determine structures. Although X-ray crystallography has been a powerful method to visualize the architecture of ABC transporters at high resolution, however, the arduous nature of getting a high-quality crystal requiring large amounts of protein had been a time consuming process which can take from days to months or years depending on the target protein. It was another reason for the slow progress and lag phase in the beginning of the field. Nevertheless, advancements in cryo-EM, which requires less amount of protein and provides an opportunity to study fragile and flexible membrane proteins in the native environment, intrigued experts to use it as a prime tool to determine structures and revolutionize the field [27]. The X-ray crystallography of membrane proteins was impeded by a major obstacle of sample and conformational heterogeneity which has been solved computationally in cryo-EM [9]. At the same time, robust membrane mimicking artificial systems (amphipols, nanodiscs, peptidiscs, salipro, styrene-maleic acid copolymers, and native nanodiscs) for reconstituting membrane proteins especially for cryo-EM were developing [28]. Applicability of cryo-EM in the field of ABC transporters not only allowed us to discover structures for unknown ABC transporters but also let us explore detailed mechanistic conformational landscape of individual ABC transporters at high resolution [29]. Although the cryo-EM technique was applied later in the field [30, 31], it has already determined 58% of structures and is somewhat taking over X-ray crystallography which contributed to a similar proportion (42%) of structures in the field (Figure 4(b) inset). Regardless, X-ray crystallography is still being in use to determine structures of ABC transporters and contributing to the field. Therefore, both techniques are continuously being applied to shed light on the comprehensive understanding of the field of ABC transporters.

Similar timeline distributions with respect to the resolution and fold of the structure were also studied. Resolution is an important aspect of the structure and defines the confidence in the location of atoms in the structure. The resolution range for a majority of the structures (62%) from the beginning of the field to the present is 3.0-3.9 Å (Figures 4(c) inset and 4(e)). As the field was started, structures of BtuCD and Sav1866 were determined at the resolution of 3.2 Å and 3.0 Å, respectively, using X-ray crystallography. However, in 2007, the range of resolution for structures varied between 2.8 Å and 5.5 Å. The proportion of structures for resolution ranges, 2.0-2.9 Å and 4.0-4.9 Å, is similar, i.e., 17%. (Figure 4(c) inset). While the resolution range existed in 2.0-2.9 Å in earlier years, but extended to 4.0-4.9 Å in later years (Figures 4(c)), which could be due to the inherent nature of structural tools being used to determine these structures in these time periods. Majority of the structures in resolution ranges 2.0-2.9 Å and 4.0-4.9 Å were determined via X-ray crystallography and cryo-EM, respectively (Figure 4(e)). So far in the field, the highest resolution structure was determined at 1.9 Å for CmABCB1 (PDB ID: 6A6M) using X-ray crystallography in 2019. Therefore, the field lost a bit of resolution during its shift from X-ray crystallography to cryo-EM. Nevertheless, further technological advances and accelerated data acquisition capabilities with improved sample quality provides routine application of cryo-EM for high-resolution structures. High-resolution structures not only allow the visualization of individual atoms including solvent molecules in proteins but also allow the detailed understanding of coordination between substrate and transporter, confident model building of glycan chains, and local conformational changes in amino acids from different conformations [32]. Improved visualization of these features and aspects in transporters not only provided comprehensive understanding of mechanism and structure-function relationships in ABC transporters but also opened new avenues for future structure-based drug designing for therapeutic sciences [33, 34]. Furthermore, the timeline distribution of structures of ABC transporters with respect to defined folds showed their emergence over time (Figure 4(d), Figure S2). For example, only type I, II, and IV folds were known in the beginning of field. Type III fold or ECF transporters were discovered in 2013, followed by discovery of type V in 2015 and VI and VII in 2017 (Figure 4(d)). Type IV fold, well represented in prokaryotes as well as eukaryotes, is the most abundant fold with 53% of the total proportion of structures among ABC transporters (Figures 4(d) inset and 4(f)). Type I fold, mainly represented in prokaryotes, has the second highest proportion (17%) of structures in the field (Figures 4(d) inset and 4(f)). Analysis of unique ABC transporters also follow similar trends (Figure S2, Table 2).

**Table 2:** Folds for unique ABC transporters.

Fold	Unique ABC transporters	Total number of ABC transporters with each fold
I	Maltose transporter, ModBC, MetNI, Art(QN)_2_, AlgM1M2SS, MLA complex, LpqY-SugABC, OpuA	8
II	BtuCD, H1470/1, HmuUV, BhuUV, PmtCD, PsaBC	6
III	LbECF, CbiMNOQ	2
IV	Sav1866, MsbA, P-glycoprotein, TM287/288, ABCB10, Atm1, McjD, PCAT1, PglK, TAP1/TAP2, CFTR, TmrAB, MRP1, AaPrtD, SUR, ABCD4, ABCB4, Rv1819v, IrtAB, ABCB11, YbtPQ, ABCB6, ABCB8, BmrCD, BmrA, AtAtm3, Ycf1p, ABCD1	28
V	ABCG5/ABCG8, ABCG2, ABCA1, WzmWzt, TarGH, ABCA4, ABCG1, Pdr5, ABCA3	9
VI	LptB_2_FG	2
VII	MacB, MacAB, LolCDE	3

## 6. Unique Sources Used for Structually Studying ABC Transporters

**Figure 5:**
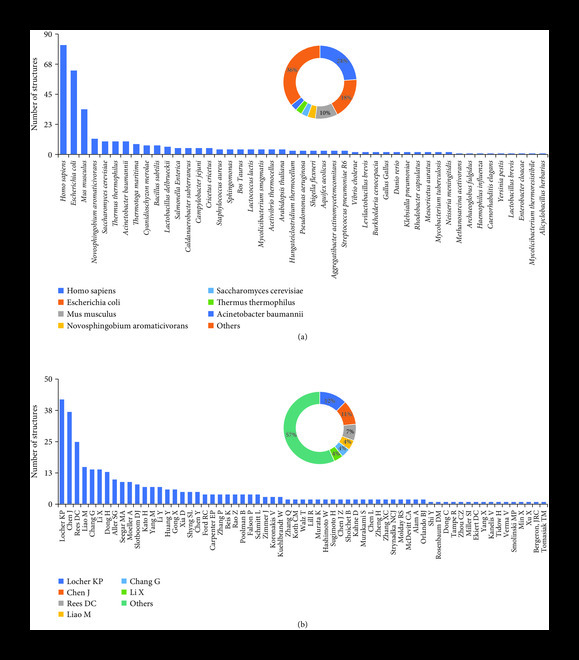
(a) Number of ABC transporters from different organisms. (b) Number of structures determined by different group leaders in the field of structural biology of ABC transporters. Insets are the pie chart with percentages of the same distribution.

Data collected on a number of structures determined from unique sources (Figure 5(a)) showed that 47 unique sources including 34 prokaryotic and 12 eukaryotic sources have been used to determine structures in the field. The three most common sources used are *Escherichia coli*, *Homo sapiens*, and *Mus musculus* whose proportions are 18%, 24%, and 10%, respectively (Figure 5(a) inset). Timeline graphs for unique sources again showed an increasing interest in exploring ABC transporters from new eukaryotic organisms in the later years (Figure S3 and Table 3) with advancements in membrane protein expression systems. The highest number of sources were explored in the year 2017 (Figure S3A, Table 3). The cumulative curve for organismal sources also shows the presence of a lag phase in the beginning followed by a continuous increasing interest in exploring new sources (Figure S3B).

**Table 3:** Unique organismal sources used for structural characterization of ABC transporters in different years.

Year	Unique organismal sources	Total number of unique sources	Prokaryotic sources	Eukaryotic sources
2002	*Escherichia coli*	1	1	0
2006	*Staphylococcus aureus*	1	1	0
2007	*Salmonella enterica, Vibrio cholera, Archaeoglobus fulgidus, Haemophilus influenzae*	4	3	0
2008	*Methanosarcina acetivornas*	1	1	0
2012	*Caenohabditis elegans, Yersinia pestis, Thermotoga maritima*	3	2	1
2013	*Mus musculus, Homo sapiens, Levilactobacilus brevis, lactobacillus brevis*	4	2	2
2014	*Novosphingobium aromaticivorans, Cyanidioschyzon merolae, Saccharomyces cerevisiae*	3	1	2
2015	*Caldanaaerobacter subterranean, Hungateiclostridium thermocellum, Campylobacter jejune, Sphingomonas*	4	4	0
2016	*Lactobacillus delbrueckii, Burkholderia cenocepacia, danio renio*	3	2	1
2017	*Klebsialla pneumonia, Pseudomonas aeruginosa, Thermus thermophilus, Bos taurus, aquifer aeolicus, Aggregatibacter actinomycetemic omitans, Acinetobacter baumanni, Rhodobacter capsulatus*	8	7	1
2018	*Gallus gallus, Streptococcus pneumonia R6, Mesocricetus auratus*	3	1	2
2019	*Shigella flexneri, Enterobacter cloacae, Cricetus cricetus*	3	1	2
2020	*Mycobacterium tuberculosis, Mycolicibacterium thermoresistible, Mycolicibacterium smegmatis, Lactococcus lactis, Alicyclobacillus herbarious*	5	5	0
2021	*Neisseria meningitidis*	1	1	0
2022	*Acetivibrio thermocellus, Bacillus subtilis, Arabidopsis thaliana*	3	2	1
		47	34	12

## 7. Group Leaders in Structural Biology of ABC Transporters

As the expression systems and structural tools were advancing and revolutionizing the field, an accruing interest of new incoming group leaders in the field and their contribution should also be acknowledged (Figure 5(b), Table 4). The last 20 years of field of structural biology of ABC transporters has attracted 65 group leaders. Kasper Locher, Jue Chen, and Douglas C Rees have been prominently leading the field (Figure 5(b) inset). Since 2006, Kasper Locher and his colleagues started with Sav1866 structure and have determined at least 42 structures in the field which has helped in investigating mechanisms of ABC transporters relevant to cellular physiology (Figure 5(b), Table 4). Jue Chen entered the field in 2007 with maltose transporter and has determined 37 structures at present (Table 4). Douglas C Rees in collaboration with Kasper Locher initiated the field of structural biology of ABC transporters by determining the first structure of BtuCD in 2002 and, since then, has contributed at least 25 structures in the PDB database (Table 4). Besides these senior investigators in the field, young investigators like Geoffrey Chang, Xiaochun Li, Haohao Dong, and Maofu Liao also showed their dedication by contributing more than 10 structures in the expanding field of ABC transporters (Figure 5(b), Table 4). Amazingly, a number of new investigators are entering the field of structural biology of ABC transporters and again the list is following the exponential phase like new organismal sources (Figure S4).

**Table 4:** Unique group leaders joining the field of structural biology of ABC transporters in every year.

Year	Unique group leaders	Total number of unique group leaders
2002	Rees DC	1
2006	Locher KP	1
2007	Chen J, Chang G	2
2012	Seegar MA	1
2013	Carpenter EP, Shi Y, Zhang P	3
2014	Aller SG, Kato H, Lill R, Beis K	4
2015	Yang M, Murata K	2
2016	Slotboom DJ, Rosenbaum DM, Sugimoto H	3
2017	Liao M, Xia D, Hashimoto W, Dong C, Huang Y, Tampe R, Zimmer J, Koronakis V, Murakami S, Gong X	10
2018	Koth CM, Ford RC, Chen JZ, Zhou CZ, Chen L	5
2019	Zhang Q, Shoichet B, Dong H, Kahne D, Moeller A, Shyng SL, Miller SI, Chen Y	8
2020	Walz T, Ekiert DC, Li Y, Bergeron JRC, Zheng H, Zhang XC, Rao Z, Poolman B, Strynadka NCJ	9
2021	Yang X, Kanelis V, Tidow H, Molday RS, Schmitt L, Tomasiak TM, McDevitt CA,	7
2022	Verma V, Smolinski MP, Kuehlbrandt W, Min X, Xu X, Li X, Falson P, Alam A, Orlando BJ	9

## 8. Impact of Published Structures in the Field of ABC Transporters

Exploding field of the structural biology of ABC transporters with a number of deposited structures in the PDB database is significantly impacting the field by expanding our understanding towards structure-function relationships and informing critical aspects of mechanism in these transporters [4]. As mentioned before, these structures contributed towards understanding the architecture of these ABC transporters and establishing a classification system for different folds [12]. On the other hand, these structures in different conformational states for various transporters not only contributed towards gaining an insight into detailed understanding of mechanisms in individual ABC transporters but also defined the general alternating access mechanism in the family [35]. Furthermore, structures of these ABC transporters in the presence of their ligands not only helped in understanding detailed enzymatic catalytic mechanism for ATP hydrolysis and its coupling with the solute transport [36] but also in deducing their substrate specificity and differentiating between substrates and inhibitors based on the induced conformational change [37]. The detailed insights for the substrate binding site in ABC transporters has also contributed towards applying the knowledge for structure-based drug designing [38] and therefore, the design of dual inhibitors to improve the bioavailability of drugs [39]. These studies illustrate the use of structural biology of ABC transporters in therapeutic sciences. The field is still calling the prospect of usage of these structures with respect to understanding their role in physiological processes which may involve combining structural tools with cell biology tools.

## 9. Conclusions and Future Directions

This article provides an overview of the revolutionizing field of structural biology of ABC transporters. The data regarding structures of ABC transporters was collected from the PDB database and various trends with respect to sources, resolution, fold, structural tools, and group leaders were studied to understand the continuously growing nature of the field. In the nutshell, this study exclusively prepares the platform to build a focused database on structurally studied ABC transporters. It evidences the emergence of different structural folds during these years and their distribution among prokaryotes and eukaryotes. The study recognizes contributions of advancements in cryo-EM and mammalian expression systems and accruing interest of emerging group leaders in the field. Follow-up study on meta-analysis of studies on the utility of these structures in other scientific areas will be useful to understand the impact of the structural biology of ABC transporters. Furthermore, the structural alignment of these structures from different sources can deduce the relationships between structure, function, and evolution in this family.

## Data Availability

Raw data is available on request.

## Supplementary Materials

Supplementary Materials Figure S1: (A) number of unique ABC transporters structurally studied every year and (B) the cumulative growth of structurally studied ABC transporters follows exponential phase. Figure S2: (A) number of structurally studied unique ABC transporters with different folds, (B) their distribution in pie chart, (C) their timeline, and (D) their cumulative growth over years. Different categories are labeled and colored accordingly in the graph. Pie charts represent percentages of these distributions. Figure S3: (A) number of unique organismal sources used every year and (B) their cumulative growth over years to structurally study ABC transporters. Blue and orange represent the distribution of prokaryotic and eukaryotic sources, respectively. Figure S4: (A) number of group leaders entering the field of structural biology of ABC transporters every year and (B) their cumulative growth is following the exponential curve.
