# Supplementary material for: Meta-Analysis of the Expansion in the Field of Structural Biology of ABC Transporters
Source: Biodes Res. 2022 Sep 8;2022:9806979. doi: 10.34133/2022/9806979 (PMC10521687; doi:10.34133/2022/9806979)
Supplement: Supplementary Materials — Figure S1: (A) number of unique ABC transporters structurally studied every year and (B) the cumulative growth of structurally studied ABC transporters follows exponential phase. Figure S2: (A) number of structurally studied unique ABC transporters with different folds, (B) their distribution in pie chart, (C) their timeline, and (D) their cumulative growth over years. Different categories are labeled and colored accordingly in the graph. Pie charts represent percentages of these distributions. Figure S3: (A) number of unique organismal sources used every year and (B) their cumulative growth over years to structurally study ABC transporters. Blue and orange represent the distribution of prokaryotic and eukaryotic sources, respectively. Figure S4: (A) number of group leaders entering the field of structural biology of ABC transporters every year and (B) their cumulative growth is following the exponential curve. [file 9806979.f1.pdf]

Meta analysis of the expansion in the field of structural biology of ABC transporters

Soomi Kim[1,2#], Teena Bajaj[3#] Cole Chabon[2#], Eric Tablante[2], Tatyana Kulchinskaya[1,2], Tae Seok Moon[4], Ruchika Bajaj[5*]
# Authors contributed equally

1. Stem Cell Technology Certificate Program, City College of San Francisco
2. Biotechnology Certificate Program, City College of San Francisco
3. Comparative Biochemistry Program, University of California Berkeley
4. Department of Energy, Environmental and Chemical Engineering, Washington University in St. Louis
5. Department of Bioengineering and Therapeutics Sciences, University of California San Francisco

*Correspondence should be addressed to phd.ruchika@gmail.com

Figures S1 - S4

Figure S1: [A] Number of unique ABC transporters structurally studied every year and [B] the cumulative growth of structurally studied ABC transporters follows exponential phase.

Figure S2: [A] Number of structurally studied unique ABC transporters with different folds, [B] their distribution in pie chart [C] their timeline and [D] their cumulative growth over years. Different categories are labeled and colored accordingly in the graph. Pie charts represent percentages of these distributions.

Figure S3: [A] Number of unique organismal sources used every year and [B] their cumulative growth over years to structurally study ABC transporters. Blue and Orange represent the distribution of prokaryotic and eukaryotic sources respectively.

Figure S4: [A] Number of group leaders entering the field of structural biology of ABC transporters every year and [B] their cumulative growth is following the exponential curve.

Figure S1

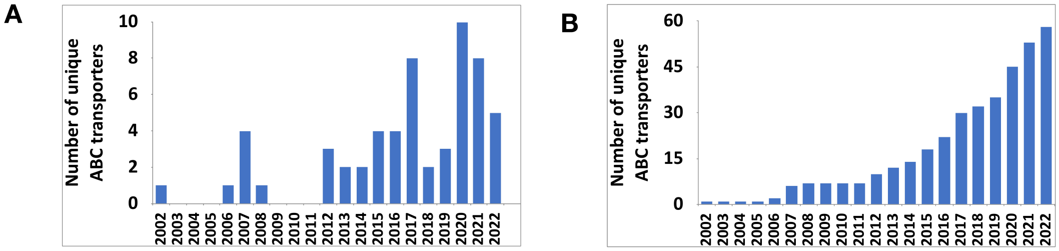

Figure S2

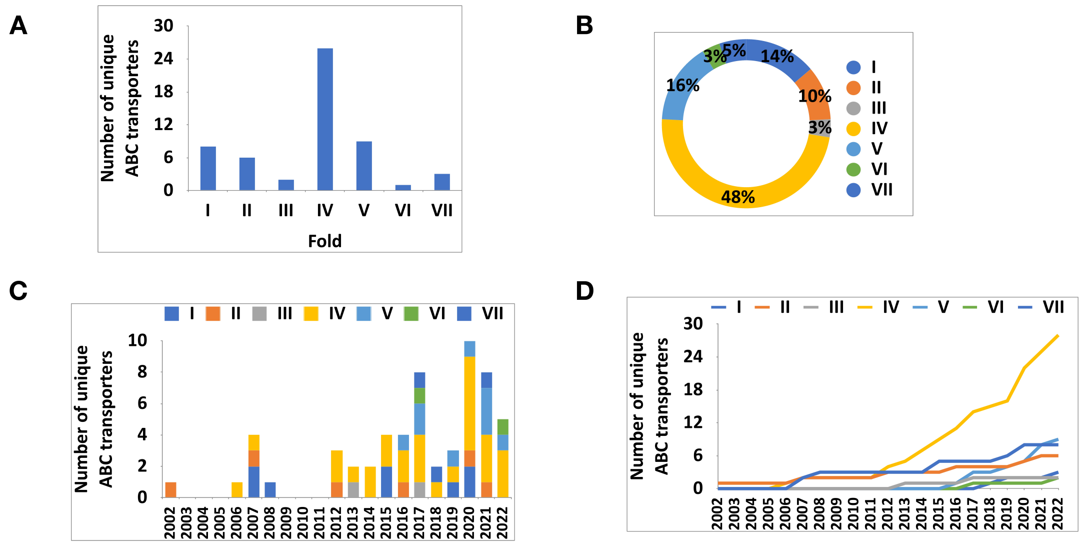
A
Number of unique ABC transporters
30
24
18
12
6
0
I II III IV V VI VII
Fold
B
14%
10%
3%
48%
16%
3%
5%
I II III IV V VI VII
C
I II III IV V VI VII
Number of unique ABC transporters
10 8 6 4 2 0
2002 2003 2004 2005 2006 2007 2008 2009 2010 2011 2012 2013 2014 2015 2016 2017 2018 2019 2020 2021 2022
D
I II III IV V VI VII
Number of unique ABC transporters
30 24 18 12 6 0
2002 2003 2004 2005 2006 2007 2008 2009 2010 2011 2012 2013 2014 2015 2016 2017 2018 2019 2020 2021 2022

Figure S3

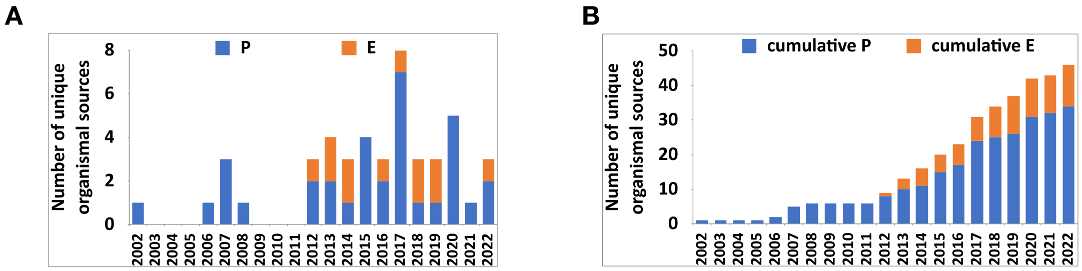
A
B
Number of unique organismal sources
P
E
cumulative P
cumulative E
0
2
4
6
8
10
20
30
40
50
2002
2003
2004
2005
2006
2007
2008
2009
2010
2011
2012
2013
2014
2015
2016
2017
2018
2019
2020
2021
2022

Figure S4

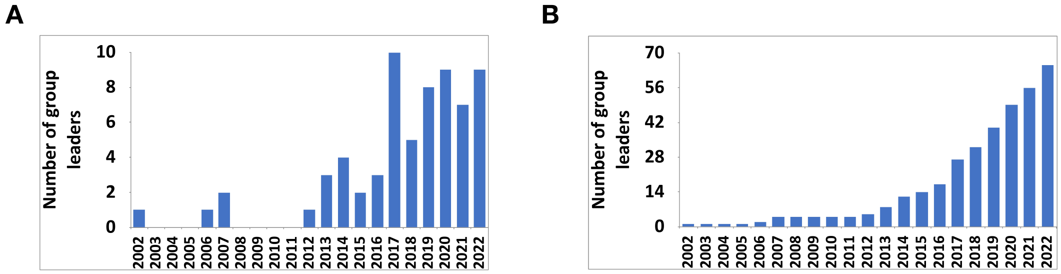
A
Number of group leaders
10
8
6
4
2
0
2002 2003 2004 2005 2006 2007 2008 2009 2010 2011 2012 2013 2014 2015 2016 2017 2018 2019 2020 2021 2022
B
Number of group leaders
70
56
42
28
14
0
2002 2003 2004 2005 2006 2007 2008 2009 2010 2011 2012 2013 2014 2015 2016 2017 2018 2019 2020 2021 2022
